# A Fast and Easy ATP-Based Approach Enables MIC Testing for Non-resuscitating VBNC Pathogens

**DOI:** 10.3389/fmicb.2019.01365

**Published:** 2019-06-14

**Authors:** Christian Robben, Anna Kristina Witte, Dagmar Schoder, Beatrix Stessl, Peter Rossmanith, Patrick Mester

**Affiliations:** ^1^Christian Doppler Laboratory for Monitoring of Microbial Contaminants, Department of Farm Animal and Public Health in Veterinary Medicine, Institute for Milk Hygiene, Milk Technology and Food Science, University of Veterinary Medicine, Vienna, Austria; ^2^Department of Farm Animal and Public Health in Veterinary Medicine, Institute of Milk Hygiene, Milk Technology and Food Science, University of Veterinary Medicine, Vienna, Austria

**Keywords:** viable but non-culturable (VBNC), antibiotic resistance, food safety, antimicrobial tolerance, minimum inhibitory concentration, metabolic activity

## Abstract

Many bacteria enter the viable but non-culturable (VBNC) state to maximize resources and increase their tolerance to harmful conditions to cope with environmental stress, which has been described for a plethora of important human and foodborne pathogens. VBNC pathogens can potentially present a serious risk to human health as they are invisible to routine microbiological culture-based methods. Of high importance is the increased tolerance to antibiotics or disinfectant measures while in the VBNC state. The greatest remaining challenge for such investigations is the lack of an appropriate, cost-effective multi-species screening method due to experimental constraints. In this study, we investigated if *de novo* ATP production of cells in the VBNC state is a suitable indicator for overall cell viability that can be utilized to determine the minimum ATP inhibitory concentration (MAIC) of antibiotics and other antimicrobials. To validate this approach, heat-stress time-kill experiments were performed with both culturable and VBNC cells. We developed a comprehensive experimental setup and demonstrated the applicability of this VBNC–MIC assay for testing the tolerance of 12 strains of 4 important bacterial species (*Escherichia coli*, *Bacillus cereus*, *Pseudomonas aeruginosa*, and *Listeria monocytogenes*) in the VBNC state to eight important antimicrobials including four different antibiotics. We confirmed that bacteria in the VBNC state were resistant to all tested antibiotics (ampicillin, imipenem, ciprofloxacin, and gentamicin) and additionally insensitive to disinfectants (benzalkonium chloride and trioctylmethylammonium chloride) and preservatives (bronopol and sodium azide). These data emphasize the need for further research regarding the characteristics of bacterial pathogens in the VBNC state and present the advantages and high-throughput capabilities of ATP determinations to investigate tolerance of VBNC pathogens to antimicrobials. The presented method should be helpful in order to identify appropriate countermeasures, treatments, or disinfectants when confronted with bacterial pathogens in the VBNC state.

## Introduction

Bacteria are exposed to environments with constantly changing conditions. To cope with these environmental stresses, many bacteria enter a viable but non-culturable (VBNC) state that results in higher tolerances to harmful conditions (Oliver, 2010; Zhang et al., 2015; Lee and Bae, 2017). The VBNC state was already discovered in 1982 by Xu et al. but has been a controversial issue and along the years it was accepted as a survival strategy of bacteria and recently has become an important part in microbial research (Pinto et al., 2015; Ayrapetyan et al., 2018). The cost of this transition is that VBNC cells lose their ability to grow on routine media, although they retain intact membranes, undamaged genetic material, and metabolic activity and can potentially revert to the active state (Oliver, 2005; Lin et al., 2017). VBNC cells form during exposure to unfavorable environmental conditions such as incompatible temperatures, pH, and starvation or by exposure to noxious chemicals (Li et al., 2014; Orruño et al., 2017). Food preservatives, cleaners, disinfectants, or chlorine are examples of substances inducing the VBNC state (Rowan, 2004; Zhang et al., 2015). However, research has shown that large populations of VBNC cells also exist in unstressed environments, which appear to be a stochastic physiological transformation to protect a subpopulation of cells from unexpected stresses (Ayrapetyan et al., 2015; Gonçalves and de Carvalho, 2016). It has also been demonstrated that VBNC bacteria are able to resist a wide range of unfavorable conditions including starvation, temperature shifts, high or low pH and salinity, oxidative stress, cleaners, antimicrobial compounds, and antibiotics (Nowakowska and Oliver, 2013; Li et al., 2014; Zhao et al., 2017; Ayrapetyan et al., 2018; Robben et al., 2018). In addition, the accumulation of several non-toxic stresses has been shown to switch pathogens into the VBNC state (Overney et al., 2017). The VBNC state of pathogens therefore has relevance to human and animal health.

Emerging bacterial resistance to antibiotics has become a global environmental problem. In contrast to culturable cells, where antibiotic resistance involves specific resistance genes, resistance of VBNC cells is essentially believed to result from a non-specific stress reaction that slows metabolic activity and thereby minimizes the consequences of otherwise unimpaired antibiotic binding (Lewis, 2006; Lin et al., 2017). Research has also shown that efflux genes are upregulated in VBNC bacteria and this contributes actively to antibiotic defense (Harms et al., 2016; Pu et al., 2016; Sikri et al., 2018).

Antimicrobials are included in the range of substances that have been reported to induce the VBNC state. For example, foods treated with antimicrobials have been found generally to harbor a higher number of VBNC cells (Anvarian et al., 2016; Zhao et al., 2017). Nevertheless, published research regarding the persistence of VBNC bacteria in the presence of antimicrobials is limited. Nowakowska and Oliver (2013) showed that VBNC cells of *Vibrio vulnificus* are protected against a wide variety of stresses by resuscitating the VBNC cells following stress removal. However, from over 100 species known to enter the VBNC state, only a small number of pathogens have been successfully resuscitated (Ayrapetyan et al., 2018). To analyze VBNC bacteria that are not able to resuscitate, Lin et al. (2017) used flow cytometry to analyze the antibiotic resistance of a non-resuscitable VBNC *Escherichia coli* strain. In contrast, in culturable bacteria, antimicrobial susceptibility and resistance are generally measured by the minimum inhibitory concentration (MIC), which is the lowest concentration of an antimicrobial that prevents visible growth of a microorganism (Andrews, 2001; Li et al., 2017). Consequently, there is a need for a cost- and time-effective analysis method to investigate in more detail the tolerance of VBNC cells to antimicrobials, which is neither dependent upon bacterial resuscitation nor culture. Since all VBNC bacteria have in common the need to remain metabolically active, we selected ATP generation of VBNC cells as an indicator for vitality during and after experimental stress exposure. After confirmation of the VBNC state by investigating metabolic activity and membrane integrity, we therefore exposed VBNC pathogens to serial dilutions of antibiotics, disinfectants, and preservatives, comparable to MIC testing for culturable cells, and determined the VBNC–MIC by investigating the *de novo* ATP generation of those cells during stress exposure and compared the outcome to culturable cells.

This approach permits us to investigate the VBNC state of pathogens, such as *Listeria monocytogenes*, that have been shown to resuscitate only under very specific conditions, without the need for novel technology. With this approach we established a protocol to determine the MIC of antibiotics and antimicrobials on VBNC cells and thereby their susceptibilities, by using the BacTiter-Glo^TM^ Microbial Cell Viability Assay.

## Materials and Methods

### Bacterial Strains and Culture Conditions

This study employed four strains of *L. monocytogenes*: 3253 (sequence type-ST121) (Schmitz-Esser et al., 2015), EGDe (ST35), QC1 (ST403), and ScottA (ST290); three strains of *E. coli*: BW25113, JW5503, and ATCC25922; three strains of *Pseudomonas aeruginosa*: ATCC10145, K71, and K73; and two strains of *Bacillus cereus*: ATCC11778 and KSS. Bacterial strains are part of the collection of bacterial strains at the Institute of Milk Hygiene, Milk Technology and Food Science, University of Veterinary Medicine, Vienna, Austria.

Overnight cultures were prepared by selecting a single colony of the respective strain and growing it in a 9 ml of fresh brain heart infusion (BHI) broth supplemented with yeast extract in a shaking incubator. *L. monocytogenes*, *E. coli*, and *B. cereus* strains were incubated at 37°C while *Pseudomonas* strains were incubated at 30°C.

### Minimum Inhibitory Concentration (MIC) Assessment of Culturable Cells

Minimum inhibitory concentrations of the respective antimicrobial compounds were calculated by applying the serial twofold dilution microtiter plate method in BHI medium. In order to create a constant cell status for each experiment, 1 ml aliquots of the respective overnight cultures were transferred into 9 ml of fresh BHI medium and incubated until it reached an OD_600_ of 0.6. This ensured that cells were in a logarithmic growth phase. Subsequently, each well, which contained a serially diluted antimicrobial (dilution 1:2), was inoculated with 5 × 10^5^ CFU/ml of the respective bacterial cells. After bacterial inoculation, the 96-well microtiter plates (Corning B.V. Life Sciences, Amsterdam, Netherlands) were measured at 610 nm wavelength in a TECAN F100 microplate reader (Tecan Austria GmbH, Groeding, Austria) to monitor any possible interference by the compounds at the given wavelength. The microtiter plates were then incubated for 24 h at 30°C for *Pseudomonas* strains and at 37°C for all other strains. Afterward bacterial growth was assessed by measuring absorbance at 610 nm. The MIC was defined as the lowest concentration of the antimicrobial where no bacterial growth could be measured after 24 h. Results are displayed as mean MIC, including upper and lower limits, of at least three experiments performed on different days.

### Induction and Confirmation of the VBNC State

Induction and confirmation of the VBNC state was performed according to Robben et al. (2018). One milliliter of early log phase bacteria (OD_600_: 0.6) was centrifuged for 5 min at 8,000 ×*g*. The pellet was resuspended in 1 ml of the respective treatment conditions (Table 1) and incubated for 1 h at room temperature (RT) (Robben et al., 2018). After incubation, samples were centrifuged for 5 min at 8,000 ×*g* and pellets washed with 1 ml of 1× phosphate buffer solution (PBS). Cells were resuspended in 1 ml BHI medium and maintained at their respective incubation temperature.

**Table 1:** VBNC induction conditions.

*L. monocytogenes*	1% Lutensol XP30 + 1 M MgCl_2_
*E. coli*	1% Lutensol XP30 + 1 M K_2_CO_3_
*B. cereus*	1% Lutensol XP30 + 2 M MgCl_2_
*P. aeruginosa*	1% Lutensol XP30 + 2 M MgCl_2_


*Induction of the VBNC state was performed by incubation of culturable cells with the respective combination of detergent and salt for 1 h.*

**FIGURE 1:**
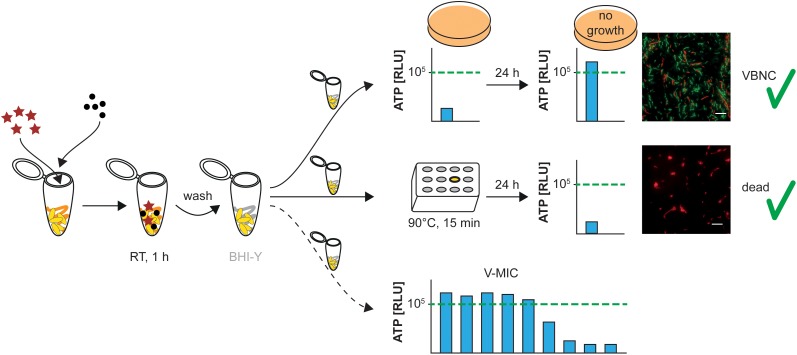
Workflow of VBNC–MIC assay. VBNC state is induced by a specific combination of detergent (stars) and salt (dots); after induction, cells are washed and resuspended in fresh BHI medium; positive control: untreated VBNC cells confirmed by ATP measurements and *Bac*Light viability assay; negative control: VBNC cells killed at 90°C for 15 min; MAIC of antimicrobials is determined by measuring *de novo* ATP production of VBNC cells exposed to the respective antimicrobial, viability marker: 10^5^ RLU threshold. Scale bar: 50 μm.

To confirm VBNC induction, culturability and ATP production were tested directly and 24 h after resuspending cells in fresh BHI medium (Figure 1). Corresponding to Robben et al. (2018) we set the threshold of 10^5^ RLU for *de novo* ATP production within 24 h, which is an empirical value derived from the previous publication, to make sure that sufficient cells entered the VBNC cells with regard to further experiments. Testing culturability after 24 h also served as a control in order to exclude possible regrowth of remaining culturable cells in the respective samples. Culturability testing was performed by plating 10 μl of the culture onto a tryptone soya agar (TSA) plate and overnight incubation at 37°C.

### Metabolic Activity of Non-culturable Bacteria

To investigate the metabolic activity of non-culturable cells, ATP was quantified using the BacTiter-Glo^TM^ Microbial Cell Viability Assay (Promega, Madison, WI, United States). Therefore, 100 μl of bacterial culture was mixed with 100 μl luciferase reagent and incubated for 10 min at RT in the dark. Luminescence was recorded in relative light units (RLUs) using the Tecan F100 microplate reader (Männedorf, Switzerland). BHI medium was used as negative control.

### Establishing the Experimental Setup to Determine the Minimum ATP Inhibitory Concentration (MAIC)

To determine the minimum ATP inhibitory concentration (MAIC), respective VBNC induction conditions for each bacterial species had to be determined by preceding screening (Robben et al., 2018). In principle, the respective bacterial strains (*L. monocytogenes* EGDe, ScottA, QC1, and MFPT3; *Escherichia coli* BW, ATCC, and JW; *B. cereus* KSS and ATCC; *Pseudomonas aeruginosa* ATCC, K71, and K73) were exposed to different combinations of non-ionic detergents and inorganic salts for 1 h (Table 1). After incubation, a successful induction of the VBNC state was determined as follows. Cells were confirmed to have entered the VBNC state if they: (i) lost their ability to grow on non-selective agar plates, (ii) could produce *de novo* ATP over a level of 10^5^ RLU within 24 h without regaining their culturability, but (iii) could be killed by incubation at 90°C for 15 min (Figure 1).

In addition to the preceding screening, the optimal induction conditions for each strain and the subsequent incubation conditions for optimal *de novo* ATP generation had to be evaluated for each strain individually. For this purpose, three different cell concentrations of each strain (OD_600_ of 0.2, 0.4, and 0.6) as well as three different incubation temperatures (RT, 30, and 37°C) were tested. To exclude ATP production by regrown cells or resuscitation, 10 μl of each individual sample was plated onto TSA plates. All tests were done in triplicates. Conditions leading to the maximum *de novo* ATP production were used for further experiments.

### Temperature-Dependent Time-Kill Experiment to Evaluate *de novo* ATP Production as Viability Indicator

After setting the optimal framework conditions, a series of temperature-dependent time-kill experiments were conducted to evaluate *de novo* ATP production as an indicator of overall bactericidal effect of a defined stress. In these experiments, the CFU/ml of a *L. monocytogenes* EGDe culture was measured over a period of 180 min when exposed to three different incubation temperatures (50, 60, and 70°C). The plate count results of the culturable cells were compared to the *de novo* ATP production of VBNC cells after 24 h incubation in BHI medium. This was done in triplicates.

### Assessment of the Minimum ATP Inhibitory Concentration of VBNC Cells (VBNC–MIC)

Before determination of the MAIC, the general induction procedure was performed as described above. After induction, the cells were pelleted via centrifugation (5 min, 8,000 ×*g*) and washed once with PBS to remove the induction solution, before resuspending the bacterial pellet in fresh BHI medium (final concentration: ∼5 × 10^8^ cells/ml). In order to make sure that the cells are really in the VBNC state, a part of the VBNC suspension was aliquoted into a separate vial and subsequently heated at 90°C for 15 min to kill all cells reliably to serve as a (negative) control. Subsequently, 10 μl of each cell suspension was plated onto TSA plates, to check for remaining culturable cells or resuscitation, and the initial ATP concentration in each suspension was measured to determine the background level released from suspended cells (Figure 1).

Again, to determine the MAICs of the respective antimicrobial compounds, the serial twofold dilution was applied in a 96-well microtiter plate with BHI medium. Subsequently a 100 μl aliquot of the VBNC cell suspension was added to the microtiter plate to give the final desired concentration. The suspensions were then incubated at 37°C for 24 h, after which the ATP concentration was determined. The MAIC was identified by the threshold whereby *de novo* ATP production with increasing antimicrobial concentration dropped below 10^5^ RLU.

### LIVE/DEAD^TM^ *Bac*Light^TM^ Bacterial Viability Fluorescence Microscopy

To investigate if cell membranes of non-culturable bacteria remained intact after exposure to the respective antimicrobial treatment, we used the LIVE/DEAD^TM^ *Bac*Light^TM^ bacterial viability kit (Molecular Probes, Eugene, OR, United States). Therefore, 100 μl of the bacterial sample was removed from the respective wells, centrifuged at 8,000 ×*g* for 5 min, washed with PBS twice, and diluted 1:100. Then 1.5 μl of SYTO 9 dye and 1.5 μl of propidium iodide were added to the sample, followed by vortexing and 15 min incubation in the dark. After incubation, cells were trapped with a 0.2-μm polycarbonate membrane filter (Sterlitech, Kent, WA, United States), which were subsequently placed between a slide and a coverslip and used for fluorescence microscopy. Viable cells with intact membranes will appear green by fluorescent SYTO 9, which permeates intact or damaged plasma membrane, while propidium iodide can only permeate damaged and disrupted plasma membranes and competes with SYTO 9 for DNA-binding sites. Therefore, cells with intact membranes show green fluorescence while cells with damaged membranes appear with red fluorescence. Those were considered to be dead. Pictures were taken with the Zeiss Observer Z1 inverted widefield microscope and ZEN 2012 (blue edition) software (Oberkochen, Germany).

## Results

### Setup to Determine the Minimum ATP Inhibitory Concentration

To determine the MAIC, VBNC induction conditions for each bacterial species (*L. monocytogenes* EGDe, ScottA, QC1, and MFPT3; *Escherichia coli* BW, ATCC, and JW; *B. cereus* KSS and ATCC; and *Pseudomonas aeruginosa* ATCC, K71, and K73) had to be evaluated by a preceding screening, as described by Robben et al. (2018). The VBNC state was confirmed if cells lost the ability to grow on non-selective agar plates, could produce *de novo* ATP over a level of 10^5^ RLU within 24 h without regaining their culturability, and could be killed by incubation at 90°C for 15 min (Figure 1).

As a result of the preceding screening, a non-ionic detergent–salt combination was determined for each of the 12 bacterial strains, which led to stable VBNC induction (Table 1). Nevertheless, as induction of the VBNC state is not a 100% reliable process the respective VBNC confirmation and controls, comparison of BacLight viability assay, and ATP measurements of VBNC and dead bacteria, as described in Figure 1, were performed for each corresponding experiment.

**FIGURE 2:**
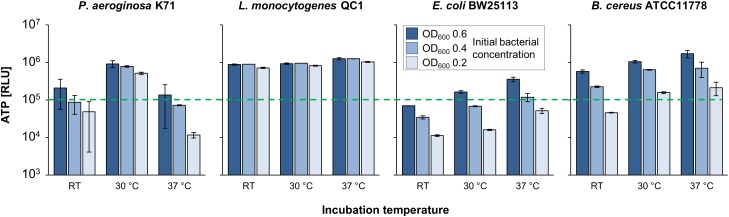
VBNC incubation setup for optimum framework conditions. *De novo* ATP production [relative light units (RLU)] of VBNC cells was monitored at room temperature (RT), 30, and 37°C at three different optical densities (OD_600_: 0.2, 0.4, and 0.6) with different bacterial strains.

For clarity purposes, only exemplary results for one representative strain per bacterial species are depicted in Figure 2, due to the fact that no differences between the respective strains were found (data not shown).

The highest *de novo* ATP production after 24 h incubation in BHI was found to be at the respective optimal growth temperature and at the highest cell density of each strain (Figure 2). Based on these results, from this point forward incubation of the culture after VBNC induction was performed at 37°C for *E. coli*, *L. monocytogenes*, and *B. cereus*. For *P. aeruginosa* strains, the incubation temperature was adapted to their optimal temperature of 30°C, as it resulted in higher ATP production compared to incubation at RT or 37°C. Cultures were used with an optical density of 0.6 (Figure 2).

### Loss of *de novo* ATP Production of Cells in the VBNC State Is a Suitable Indicator of Killing Efficiency

**FIGURE 3:**
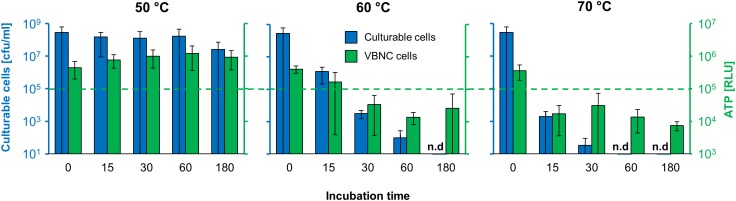
Evaluation of the VBNC–MIC assay by temperature-dependent time-kill curves. *De novo* ATP production (green bars) of VBNC *L. monocytogenes* was determined and compared to plate count (blue bars) of culturable *L. monocytogenes* after incubation at 50, 60, and 70°C over a period of 180 min; n.d., not detectable.

After setting the optimal framework conditions, temperature-dependent time-kill experiments were conducted to evaluate *de novo* ATP production as an indicator of the overall bactericidal effect of a defined stress. The plate count results after 24 h of incubation are presented as blue bars in Figure 3. The identical experimental approach was used with *L. monocytogenes* cells in the VBNC state with a subsequent measurement of *de novo* ATP production after 24 h incubation in BHI medium indicated as green bars.

After incubation at 50°C, no significant reduction in the CFU/ml was found, demonstrating the ability of *L. monocytogenes* to withstand this stress condition for at least 60 min. Similar results were also obtained for cells in the VBNC state, where no significant impact of the incubation temperature on subsequent *de novo* ATP production was found.

In the case of 60°C incubation, there was a clear time dependency in terms of reduction of living cells for both the culturable cells as well as for the VBNC cells. In the case of the culturable cells, a two log reduction was found after 15 min, while after 30, 60, and 180 min a four-log reduction (indicating disinfection efficiency) was found. In the case of the VBNC cells, *de novo* ATP production was still clearly above the 10^5^ RLU threshold after 15 min, indicating that the VBNC cells were still intact, while after 30, 60, and 180 min the number was below this threshold. Similar results were also obtained at 70°C, where both the culturable cells and the VBNC cells were reduced to a non-detectable level after merely 15 min of incubation. Overall, there was good agreement between the results for *L. monocytogenes* EGDe in either the culturable or VBNC state at each of the different incubation temperatures.

### Validation of the VBNC–MIC Assay

While the results of the time-kill heat stress experiment indicate that this method could be used for such semi-quantitative analysis, the focus of this study was to use it as a qualitative Yes/No screening method comparable to classic microbiological MIC testing.

**FIGURE 4:**
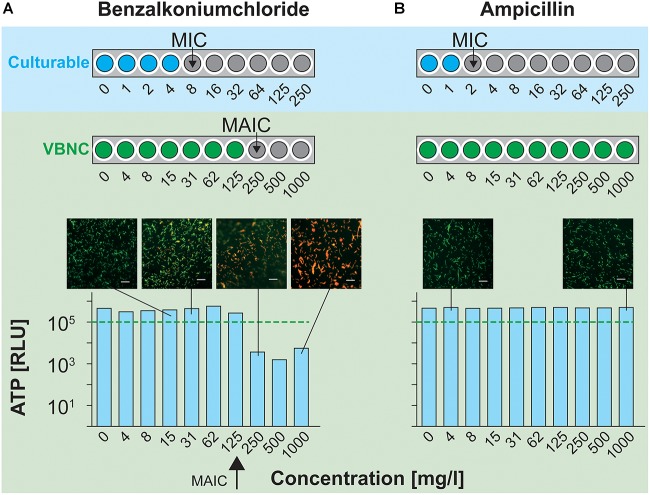
MIC determination for culturable and MAIC determination for VBNC *L. monocytogenes*. **(A)** MIC of benzalkonium chloride (BK) (blue dots) was assessed by the standard broth dilution procedure (MIC assay) and plate count method. MAIC (green dots) of BK for VBNC *L. monocytogenes* was determined by *de novo* ATP measurements (VBNC–MIC assay). The *Bac*Light viability assay was used to validate the VBNC–MIC assay. **(B)** MIC and MAIC assessment of Amp. Scale bar: 50 μm.

Therefore, an initial MIC screening of the disinfectant benzalkonium chloride (BK) and antibiotic ampicillin (Amp) on *L. monocytogenes* EGDe in the “normal” culturable state as well as the VBNC state was performed (VBNC–MIC assay). In the case of the VBNC cells, *de novo* ATP production in the presence of the respective stress was measured after 24 h of incubation and the cellular status was determined with the *Bac*Light viability assay (Figure 4).

In the case of BK, the MIC for culturable cells was 8 mg/l, while in the VBNC state a MAIC of 250 mg/l was calculated. The values determined by the ATP measurements correlated well with the cellular status determined with the *Bac*Light viability assay. At BK concentrations where *de novo* ATP production exceeded 10^5^ RLU, the majority of the cells appeared bright green, indicating intact cell membranes and overall cell viability (Figure 4A). At the respective MAIC of 250 mg/l, cells switched to appear predominantly red instead of green, indicating damaged cell membranes and thus non-viable cells.

In the case of Amp, the amount of *de novo* ATP production by *L. monocytogenes* in the VBNC state did not drop below the threshold of 10^5^ RLU for any concentration within the experimental approach (maximum tested concentration of 1,000 mg/l). In contrast, the MIC for culturable cells was 1 mg/l. These results were confirmed with the *Bac*Light assay, where VBNC cells appeared bright green, even at 1,000 mg/l (Figure 4B).

### Resistance of VBNC Cells to Antibiotics

To evaluate results obtained, we performed an extended screening of the 12 bacterial strains and determined the respective MIC and MAIC of four antibiotics (Amp, imipenem, ciprofloxacin, and gentamicin). As there were no significant differences between the different strains of the respective bacterial species, for clarity purposes only exemplary results for one representative strain per bacterial species are depicted in Table 2, and the results for each of the 12 strains are compared in Supplementary Table S1.

**Table 2:** MIC and MAIC of culturable and VBNC pathogens.

	*L. monocytogenes* QC1		*E. coli* BW25113		*B. cereus* ATCC11778		*P. aeruginosa* K71	
Antibiotics	MIC	MAIC	MIC	MAIC	MIC	MAIC	MIC	MAIC
Ampicillin	1.9 [0.9]	>1000 [0]	5.3 [1.2]	>1000 [0]	125.0 [0.0]	>1000 [0]	>250 [0]	>1000 [0]
Ciprofloxacin	13.8 [10.6]	>1000 [0]	0.3 [0.1]	>1000 [0]	0.8 [1.0]	>1000 [0]	0.4 [0.1]	>1000 [0]
Gentamicin	22.0 [5.2]	>1000 [0]	17.7 [6.7]	>1000 [0]	8.0 [0.0]	>1000 [0]	2.0 [0]	>1000 [0]
Imipenem	0.4 [0.4]	>1000 [0]	1.2 [0.3]	>1000 [0]	0.9 [0.6]	>1000 [0]	26.0 [8.7]	>1000 [0]


*Mean MIC/MAIC values (mg/l) of antibiotics after 24 h incubation at 37°C (30°C for *Pseudomonas* strains). Standard deviation is shown in brackets.*

Results clearly show that VBNC pathogens were resistant to all the antibiotics used, while culturable cells completely lost their culturability during overnight incubation in the presence of Amp, imipenem, ciprofloxacin, and gentamicin.

### Tolerance of VBNC Cells to Disinfectants and Preservatives

The MIC and MAIC were also determined for two quaternary ammonium compounds (QACs) [BK and trioctylmethylammonium (TOMA) chloride] and two preservatives (bronopol and sodium azide). As there were no significant differences between the different strains of the respective bacterial species only exemplary results for one representative strain per bacterial species are depicted in Table 3, and the results for each of the 12 strains are listed in Supplementary Table S1.

**Table 3:** MIC and MAIC of culturable and VBNC pathogens.

	*L. monocytogenes* QC1		*E. coli* BW25113		*B. cereus* ATCC11778		*P. aeruginosa* K71	
Quaternary ammoniumcompounds	MIC	MAIC	MIC	MAIC	MIC	MAIC	MIC	MAIC
Benzalkonium chloride	5.5 [1.0]	375.0 [144.3]	39.0 [12.9]	750.0 [288.7]	4.7 [2.3]	250 [0]	31.0 [0]	208.3 [72.2]
Trioctylmethylammonium chloride	1.7 [0.6]	333.3 [144.3]	6.7 [2.3]	625.0 [433.0]	0.3 [0.2]	416.7 [144.3]	16.0 [0]	416.7 [144.3]
Preservatives								
Bronopol	62.0 [0]	666.7 [288.7]	36.3 [23.5]	1000.0 [0]	8.7 [7.0]	500.0 [0]	4.0 [0]	>1000 [0]
Sodium azide	250.0 [0]	>1000 [0]	833.3 [288.7]	>1000 [0]	62.0 [0]	>1000 [0]	125.0 [0]	>1000 [0]


*Mean MIC/MAIC values (mg/l) of QACs and preservatives after 24 h incubation at 37°C (30°C for *Pseudomonas* strains). Standard deviation is shown in brackets.*

Indeed, VBNC pathogens were much more tolerant to BK and TOMA chloride as well as to the preservatives bronopol and sodium azide.

Taking *L. monocytogenes* as a representative example, it is up to 220 times more tolerant to QACs compared to its culturable form. While the averaged MIC for culturable cells was 6.9 mg/l, VBNC *L. monocytogenes* survived until a mean concentration of 381.3 mg/l. Similar results were obtained for TOMA chloride. In addition, the MIC and MAIC of the preservatives bronopol and sodium azide were tested. While the mean MIC concentration of bronopol on culturable *L. monocytogenes* was 59.5 mg/l, VBNC cells were only inactivated using a concentration exceeding 700 mg/l. Moreover, they were resistant to sodium azide, the second preservative tested. Related results were obtained for *E. coli*, *B. cereus*, and *P. aeruginosa*, where VBNC cells were distinctly more tolerant than culturable cells. Further information regarding differences within each species detailed test results and statistics can be taken from Supplementary Table S1.

## Discussion

The VBNC state is a unique survival strategy adopted by many bacteria in response to adverse environmental conditions. However, the VBNC state is a major problem when it involves pathogens, where they can evade culture-based standard detection methods and tolerate higher antibiotic and disinfectant concentrations (Ramamurthy et al., 2014). It is therefore essential to understand the characteristics of cells in the VBNC state, which unfortunately has been very difficult to investigate experimentally until now.

Therefore, the aim of this study was to develop a cost-effective, time-saving, high-throughput method for determining the tolerance of VBNC bacteria to antimicrobials based on *de novo* ATP production. ATP measurements have been successfully and routinely performed for the detection or confirmation of viable bacterial cells, including those in the VBNC state, for more than a decade (Lindback et al., 2010). ATP measurements have been successfully and routinely performed for the detection or confirmation of viable bacterial cells, including those in the VBNC state, for more than a decade. However, to the best of our knowledge, *de novo* ATP production has never been utilized as an indicator of effective bactericidal concentrations.

### Establishment of the VBNC–MIC Assay for High-Throughput MAIC Determinations

In order to develop this novel VBNC–MIC screening approach, we induced the VBNC state in 12 different bacterial strains through a combinational effect of non-ionic detergents and salts. For the VBNC–MIC assay to work, it is essential that the cells in the VBNC state are still metabolically active to produce enough ATP *de novo* within 24 h, which permits reliably differentiation of VBNC cells from dead bacteria. We found that the optimum framework conditions were an initial cell density of ∼1 × 10^8^ CFU/ml (OD_600_ of 0.6) with a subsequent 24 h incubation at the respective optimal growth temperature of each strain.

The overall reliability of the VBNC–MIC approach was confirmed by a series of time-kill experiments, where the effect of three different incubation temperatures (50, 60, and 70°C) within 3 h on *L. monocytogenes* EGDe in the culturable as well as in the VBNC state were compared. While Nowakowska and Oliver (2013) reported a higher tolerance of *V. vulnificus* to 50°C stress, we suspected that yet higher stress would eventually inactivate VBNC cells. Indeed, our results show that the course of the time-kill curves of VBNC bacteria under temperature stress was very similar to those of culturable cells. Although the use of RLUs as a quantitative measure is in this case not completely applicable, we could see semi-quantitative correlations to the course of culturable cells that confirmed that this method is reliable to answer binary-based questions. As confirmation, we double-checked the outcome with BacLight fluorescence staining and obtained corresponding results, which validated 10^5^ RLU as a reliable cut-off to distinguish between viable and dead cells. Consequently, the new VBNC–MIC approach can be used in a straightforward way to compare the tolerance of cells in the VBNC state to those in the culturable state to a defined stress. While the results of the time-kill heat stress experiments indicate that this method could be used for a semi-quantitative analysis, the focus of this study was to use it as a qualitative Yes/No screening method comparable to classic microbiological MIC testing.

With this VBNC–MIC approach, the susceptibility of four different *L. monocytogenes* strains, three *E. coli* strains, two *B. cereus* strains, and three *P. aeruginosa* strains in the exponential phase of growth and in the VBNC state, to antibiotics and antimicrobials, was tested by MAIC determination. This variety of strains and species, with a range of susceptibilities to antimicrobials in the culturable state, was used to analyze different responses of their VBNC counterparts to antimicrobial stress.

### VBNC–MIC Assay Reveals That VBNC Pathogens Withstand Antimicrobials

While previous studies were either limited to resuscitation of *V. vulnificus* or utilized more complex experimental setups using RT-qPCR or fluorescence-activated cell sorting (FACS), the results obtained in this study using the VBNC–MIC assay corroborate previous results and demonstrate the complete ineffectiveness of antibiotics to bacterial cells in the VBNC state (Lleò et al., 2003; Oliver, 2010; Nowakowska and Oliver, 2013; Lin et al., 2017). We could show that when in the VBNC state, each of 12 investigated bacterial strains became resistant to Amp, imipenem, gentamicin, and ciprofloxacin, which represent three major antibiotic classes. Additionally, the MIC for the culturable bacteria is in agreement with clinical breakpoints for bacteria, according to the European Committee on Antimicrobial Susceptibility Testing (EUCAST). Hereby, Amp represents the beta-lactam antibiotics and targets cell wall synthesis by inhibiting the enzyme transpeptidase (Bamford et al., 2017). Imipenem is also a beta-lactam but unlike Amp, it is a member of the carbapenem class and remains stable in the presence of beta-lactamase. Imipenem has been shown to activate peptidoglycan genes in *P. aeruginosa* and may contribute to VBNC induction, as increased thickness of the cell wall is characteristic for some VBNC pathogens (Davies et al., 2006; Oliver, 2010; Pasquaroli et al., 2014). Ciprofloxacin, a synthetic fluoroquinolone, inhibits cell division by inhibiting DNA gyrase and is also used for VBNC detection by direct viable counting (Ramamurthy et al., 2014). Gentamicin, an aminoglycoside that irreversibly binds to the 16S rRNA component of the 30S subunit of the bacterial ribosome and inhibits protein synthesis, was shown not only to be ineffective against VBNC pathogens but could also induce the VBNC state in culturable *P. aeruginosa* (Kohanski et al., 2010; Lee and Bae, 2018). Research shows that VBNC pathogens are protected from antibiotics by their lower metabolic activity, growth arrest, and higher efflux rates (Lin et al., 2017; Sikri et al., 2018). These findings are consistent with our results.

In addition to the antibiotics, this study also compared the susceptibility of bacteria in the VBNC state to a QAC (BK; often used in cleaning solutions and disinfectants) and an ionic liquid (TOMA chloride, a promising new class of disinfectant) (Abbaszadegan et al., 2015; Mester et al., 2016; Gundolf et al., 2018). QACs have strong bactericidal activity, depending upon their structure and length of the alkyl side chain (Ferreira et al., 2011). QACs disrupt cell walls and result in leakage of the cytoplasm, therefore exemplifying a completely different antimicrobial approach than antibiotics (Bragg et al., 2014). The results of this study show that, in the VBNC state, bacterial cells were up to 300 times more tolerant to BK and TOMA chloride compared with culturable equivalents. Possible explanations for this observed tolerance could be increased membrane thickness, reduced membrane fluidity, or increased efflux pump activity, which have all been reported for the VBNC state and each of which would reduce the effectiveness of QACs or ionic liquids (Mester et al., 2016). However, in marked contrast, Rowe et al. (1998) reported that VBNC *Campylobacter jejuni* was more sensitive to BK than culturable *C. jejuni*. As only limited research has been conducted so far on this topic, it is not clear if these differences are a result of different induction procedures, or characteristic of the antimicrobials or bacterial strains used.

Similar to BK and TOMA chloride, 12 strains in the VBNC state demonstrated higher tolerance to the preservatives bronopol (a formaldehyde releaser) and sodium azide (known to inhibit microbial growth and oxidation of manganese, iron, and sulfur by cytochrome c oxidase) compared to respective culturable cells (Wang and Levin, 2009; Kireche et al., 2011; Cabrol et al., 2017). Again, the results obtained with the VBNC–MIC approach in this study are in good agreement with previous studies that demonstrated elevated resistance of bacterial cells in the VBNC state to antibiotic treatments using different strains and methods (Nowakowska and Oliver, 2013).

This observed tolerance of bacterial cells in the VBNC state is a serious concern for human health. Cleaning and disinfection strategies are currently adapted to culturable bacteria and in light of this study are not now seen to be sufficient as a proper intervention against VBNC pathogens. From our point of view, these results have significant implications for microbiologists in all fields, especially in the context of detection, intervention, and chemotherapy when confronted with VBNC pathogens. The results show that the VBNC–MIC approach is suitable for high-throughput susceptibility screening of bacterial cells in the VBNC state. This information can be used for further research and to provide a better understanding of the underlying mechanisms regarding VBNC resistance and tolerance.

## Data Availability

All datasets generated for this study are included in the manuscript and/or the Supplementary Files.

## Author Contributions

CR, PM, AW, BS, and PR conceived and designed the experiments. CR and PM performed the experiments. CR, PM, AW, and DS analyzed the data and wrote the manuscript. BS provided the bacterial strains.

## Conflict of Interest Statement

The authors declare that the research was conducted in the absence of any commercial or financial relationships that could be construed as a potential conflict of interest.

## References

[B1] Abbaszadegan A. Nabavizadeh M. Gholami A. Aleyasin Z. S. Dorostkar S. Saliminasab M. (2015). Positively charged imidazolium-based ionic liquid-protected silver nanoparticles: a promising disinfectant in root canal treatment. *Int. Endodon. J.* 48 790–800. 10.1111/iej.12377 25269666

[B2] Andrews J. M. (2001). Determination of minimum inhibitory concentrations. *J. Antimicrob. Chemother.* 48(Suppl. 1), 5–16. 11420333 10.1093/jac/48.suppl_1.5

[B3] Anvarian A. H. P. Smith M. P. Overton T. W. (2016). The effects of orange juice clarification on the physiology of *Escherichia coli*; growth-based and flow cytometric analysis. *Int. J. Food Microbiol.* 219 38–43. 10.1016/j.ijfoodmicro.2015.11.016 26705746

[B4] Ayrapetyan M. Williams T. Oliver J. D. (2018). Relationship between the viable but nonculturable state and antibiotic persister cells. *J. Bacteriol.* 200 e249–e218. 10.1128/JB.00249-218 30082460 PMC6153661

[B5] Ayrapetyan M. Williams T. C. Baxter R. Oliver J. D. (2015). Viable but nonculturable and persister cells coexist stochastically and are induced by human serum. *Infect. Immun.* 83 4194–4203. 10.1128/IAI.00404-415 26283335 PMC4598401

[B6] Bamford R. A. Smith A. Metz J. Glover G. Titball R. W. Pagliara S. (2017). Investigating the physiology of viable but non-culturable bacteria by microfluidics and time-lapse microscopy. *BMC Biol.* 15:121. 10.1186/s12915-017-0465-464 29262826 PMC5738893

[B7] Bragg R. Jansen A. Coetzee M. van der Westhuizen W. Boucher C. (2014). Bacterial resistance to Quaternary Ammonium Compounds (QAC) disinfectants. *Adv. Exp. Med. Biol.* 808 1–13. 10.1007/978-81-322-1774-9_1 24595606

[B8] Cabrol L. Quéméneur M. Misson B. (2017). Inhibitory effects of sodium azide on microbial growth in experimental resuspension of marine sediment. *J. Microbiol. Methods* 133 62–65. 10.1016/j.mimet.2016.12.021 28039035

[B9] Davies J. Spiegelman G. B. Yim G. (2006). The world of subinhibitory antibiotic concentrations. *Curr. Opin. Microbiol.* 9 445–453. 10.1016/j.mib.2006.08.006 16942902

[B10] Ferreira C. Pereira A. M. Pereira M. C. Melo L. F. Simões M. (2011). Physiological changes induced by the quaternary ammonium compound benzyldimethyldodecylammonium chloride on *Pseudomonas fluorescens*. *J. Antimicrob. Chemother.* 66 1036–1043. 10.1093/jac/dkr028 21393196

[B11] Gonçalves F. D. de Carvalho C. C. (2016). Phenotypic modifications in *Staphylococcus aureus* cells exposed to high concentrations of vancomycin and teicoplanin. *Front. Microbiol.* 7:13. 10.3389/fmicb.2016.00013 26834731 PMC4724715

[B12] Gundolf T. Rauch B. Kalb R. Rossmanith P. Mester P. (2018). Influence of bacterial lipopolysaccharide modifications on the efficacy of antimicrobial ionic liquids. *J. Mol. Liquids* 271 220–227. 10.1016/j.molliq.2018.08.134

[B13] Harms A. Maisonneuve E. Gerdes K. (2016). Mechanisms of bacterial persistence during stress and antibiotic exposure. *Science* 354:aaf4268. 10.1126/science.aaf4268 27980159

[B14] Kireche M. Peiffer J.-L. Antonios D. Fabre I. Giménez-Arnau E. Pallardy M. (2011). Evidence for chemical and cellular reactivities of the formaldehyde releaser bronopol, independent of formaldehyde release. *Chem. Res. Toxicol.* 24 2115–2128. 10.1021/tx2002542 22034943

[B15] Kohanski M. A. Dwyer D. J. Collins J. J. (2010). How antibiotics kill bacteria: from targets to networks. *Nat. Rev. Microbiol.* 8 423–435. 10.1038/nrmicro2333 20440275 PMC2896384

[B16] Lee S. Bae S. (2017). Molecular viability testing of viable but non-culturable bacteria induced by antibiotic exposure. *Microbiol. Biotechnol.* 11 1008–1016. 10.1111/1751-7915.13039 29243404 PMC6196391

[B17] Lee S. Bae S. (2018). Molecular viability testing of viable but non-culturable bacteria induced by antibiotic exposure. *Microbiol. Biotechnol.* 11 1008–1016. 10.1111/1751-7915.13039 29243404 PMC6196391

[B18] Lewis K. (2006). Persister cells, dormancy and infectious disease. *Nat. Rev. Microbiol.* 5 48–56. 10.1038/nrmicro1557 17143318

[B19] Li J. Xie S. Ahmed S. Wang F. Gu Y. Zhang C. (2017). Antimicrobial activity and resistance: influencing factors. *Front. Pharmacol.* 8:364 10.3389/fphar.2017.00364 PMC5468421 28659799

[B20] Li L. Mendis N. Trigui H. Oliver J. D. Faucher S. P. (2014). The importance of the viable but non-culturable state in human bacterial pathogens. *Front. Microbiol.* 5:258. 10.3389/fmicb.2014.00258 24917854 PMC4040921

[B21] Lin H. Ye C. Chen S. Zhang S. Yu X. (2017). Viable but non-culturable *E. coli* induced by low level chlorination have higher persistence to antibiotics than their culturable counterparts. *Environ. Poll.* 230 242–249. 10.1016/j.envpol.2017.06.047 28662489

[B22] Lindback T. Rottenberg M. E. Roche S. M. Rorvik L. M. (2010). The ability to enter into an avirulent viable but non-culturable (VBNC) form is widespread among *Listeria monocytogenes* isolates from salmon, patients and environment. *Vet. Res.* 41:8. 10.1051/vetres/2009056 19796607 PMC2775167

[B23] Lleò M. M. Bonato B. Signoretto C. Canepari P. (2003). Vancomycin resistance is maintained in enterococci in the viable but nonculturable state and after division is resumed. *Antimicrobial. Agents Chemother.* 47 1154–1156. 10.1128/AAC.47.3.1154-1156.2003 12604561 PMC149299

[B24] Mester P. Jehle A. K. Leeb C. Kalb R. Grunert T. Rossmanith P. (2016). FTIR metabolomic fingerprint reveals different modes of action exerted by active pharmaceutical ingredient based ionic liquids (API-ILs) on *Salmonella typhimurium*. *RSC Adv.* 6 32220–32227. 10.1039/C5RA24970H

[B25] Nowakowska J. Oliver J. D. (2013). Resistance to environmental stresses by *Vibrio vulnificus* in the viable but nonculturable state. *FEMS Microbiol. Ecol.* 84 213–222. 10.1111/1574-6941.12052 23228034

[B26] Oliver J. D. (2005). The viable but nonculturable state in bacteria. *J. Microbiol.* 43 93–100. 15765062

[B27] Oliver J. D. (2010). Recent findings on the viable but nonculturable state in pathogenic bacteria. *FEMS Microbiol. Rev.* 34 415–425. 10.1111/j.1574-6976.2009.00200.x 20059548

[B28] Orruño M. Kaberdin V. R. Arana I. (2017). Survival strategies of *Escherichia coli* and *Vibrio spp*.: contribution of the viable but nonculturable phenotype to their stress-resistance and persistence in adverse environments. *World J. Microbiol. Biotechnol.* 33:45. 10.1007/s11274-017-2218-2215 28161849

[B29] Overney A. Jacques-André-Coquin J. Ng P. Carpentier B. Guillier L. Firmesse O. (2017). Impact of environmental factors on the culturability and viability of *Listeria monocytogenes* under conditions encountered in food processing plants. *Int. J. Food Microbiol.* 244 74–81. 10.1016/j.ijfoodmicro.2016.12.012 28073080

[B30] Pasquaroli S. Citterio B. Cesare A. D. Amiri M. Manti A. Vuotto C. (2014). Role of daptomycin in the induction and persistence of the viable but non-culturable state of *Staphylococcus aureus* biofilms. *Pathogens* 3 759–768. 10.3390/pathogens3030759 25438023 PMC4243440

[B31] Pinto D. Santos M. A. Chambel L. (2015). Thirty years of viable but nonculturable state research: unsolved molecular mechanisms. *Critic. Rev. Microbiol.* 41 61–76. 10.3109/1040841X.2013.794127 23848175

[B32] Pu Y. Zhao Z. Li Y. Zou J. Ma Q. Zhao Y. (2016). Enhanced efflux activity facilitates drug tolerance in dormant bacterial cells. *Mol. cell* 62 284–294. 10.1016/j.molcel.2016.03.035 27105118 PMC4850422

[B33] Ramamurthy T. Ghosh A. Pazhani G. P. Shinoda S. (2014). Current perspectives on viable but non-culturable (VBNC) pathogenic bacteria. *Front. Public Health* 2:103. 10.3389/fpubh.2014.00103 25133139 PMC4116801

[B34] Robben C. Fister S. Witte A. K. Schoder D. Rossmanith P. Mester P. (2018). Induction of the viable but non-culturable state in bacterial pathogens by household cleaners and inorganic salts. *Sci. Rep.* 8:15132. 10.1038/s41598-018-33595-33595 30310128 PMC6181970

[B35] Rowan N. J. (2004). Viable but non-culturable forms of food and waterborne bacteria: quo vadis? *Trends Food Sci. Technol.* 15 462–467. 10.1016/j.tifs.2004.02.009

[B36] Rowe M. T. Dunstall G. Kirk R. Loughney C. F. Cooke J. L. Brown S. R. (1998). Development of an image system for the study of viable but non-culturable forms of *Campylobacter jejuni* and its use to determine their resistance to disinfectants. *Food Microbiol.* 15 491–498. 10.1006/fmic.1997.0178

[B37] Schmitz-Esser S. Müller A. Stessl B. Wagner M. (2015). Genomes of sequence type 121 *Listeria monocytogenes* strains harbor highly conserved plasmids and prophages. *Front. Microbiol.* 6:380. 10.3389/fmicb.2015.00380 25972859 PMC4412001

[B38] Sikri K. Duggal P. Kumar C. Batra S. D. Vashist A. Bhaskar A. (2018). Multifaceted remodeling by vitamin C boosts sensitivity of *Mycobacterium tuberculosis* subpopulations to combination treatment by anti-tubercular drugs. *Redox Biol.gy* 15 452–466. 10.1016/j.redox.2017.12.020 29413958 PMC5975079

[B39] Wang J. D. Levin P. A. (2009). Metabolism, cell growth and the bacterial cell cycle. *Nat. Rev. Microbiol.* 7 822–827. 10.1038/nrmicro2202 19806155 PMC2887316

[B40] Zhang S. Ye C. Lin H. Lv L. Yu X. (2015). UV disinfection induces a vbnc state in *Escherichia coli* and *Pseudomonas aeruginosa*. *Environ. Sci. Technol.* 49 1721–1728. 10.1021/es505211e 25584685

[B41] Zhao X. Zhong J. Wei C. Lin C.-W. Ding T. (2017). Current perspectives on viable but non-culturable state in foodborne pathogens. *Front. Microbiol.* 8:580. 10.3389/fmicb.2017.00580 28421064 PMC5378802

