# Supplementary material for: A Fast and Easy ATP-Based Approach Enables MIC Testing for Non-resuscitating VBNC Pathogens
Source: Front Microbiol. 2019 Jun 14;10:1365. doi: 10.3389/fmicb.2019.01365 (PMC6587809; doi:10.3389/fmicb.2019.01365)
Supplement: Supplementary file 1 [file Table_1.DOCX]

| **Supplements** | | | | | | | | | | | | | | | | | | | | | | | | |
| --- | --- | --- | --- | --- | --- | --- | --- | --- | --- | --- | --- | --- | --- | --- | --- | --- | --- | --- | --- | --- | --- | --- | --- | --- |
| **Table S1: MIC and MAIC of culturable and VBNC pathogens** – Mean MIC/MAIC values (mg/l) for twelve bacterial strains with minimum and maximum values and standard deviation for antibiotics, disinfectants and preservatives after 24 h incubation at 37°C (30°C for Pseudomonas strains). | | | | | | | | | | | | | | | | | | | | | | | | |
|  | **LM EGDe** | | **LM MFPT3** | | **LM Scott A** | | **LM QC 1** | | **EC BW25113** | | **EC JW5503** | | **EC ATCC25922** | | **BC ATCC11778** | | **BC KSS** | | **PA ATCC** | | **PA K71** | | **PA K73** | |
| **Ampicillin** | **culturable** | **VBNC** | **culturable** | **VBNC** | **culturable** | **VBNC** | **culturable** | **VBNC** | **culturable** | **VBNC** | **culturable** | **VBNC** | **culturable** | **VBNC** | **culturable** | **VBNC** | **culturable** | **VBNC** | **culturable** | **VBNC** | **culturable** | **VBNC** | **culturable** | **VBNC** |
| **MIC in mg/L** | 0.4 | >1000 | 1.2 | >1000 | 2.3 | >1000 | 1.9 | >1000 | 5.3 | >1000 | 2.7 | >1000 | 4.7 | >1000 | 125.0 | >1000 | 166.7 | >1000 | >250 | >1000 | >250 | >1000 | >250 | >1000 |
| **STDEV** | 0.3 | 0.0 | 1.2 | 0.0 | 1.2 | 0.0 | 0.9 | 0.0 | 1.2 | 0.0 | 0.6 | 0.0 | 2.3 | 0.0 | 0.0 | 0.0 | 72.2 | 0.0 | 0.0 | 0.0 | 0.0 | 0.0 | 0.0 | 0.0 |
| **MAX** | 0.1 | >1000 | 0.3 | >1000 | 1.0 | >1000 | 1.3 | 0.0 | 4.0 | >1000 | 2.0 | >1000 | 2.0 | >1000 | 125.0 | >1000 | 125.0 | >1000 | >250 | >1000 | >250 | >1000 | >250 | >1000 |
| **MIN** | 0.8 | >1000 | 3.0 | >1000 | 3.0 | >1000 | 3.0 | 0.0 | 6.0 | >1000 | 3.0 | >1000 | 6.0 | >1000 | 125.0 | >1000 | 250.0 | >1000 | >250 | >1000 | >250 | >1000 | >250 | >1000 |
| **Ciprofloxacin** |  |  |  |  |  |  |  |  |  |  |  |  |  |  |  |  |  |  |  |  |  |  |  |  |
| **MIC in mg/L** | 5.5 | >1000 | 8.5 | >1000 | 7.3 | >1000 | 13.8 | >1000 | 0.3 | >1000 | 0.2 | >1000 | 0.2 | >1000 | 0.8 | >1000 | 0.2 | >1000 | 0.1 | >1000 | 0.4 | >1000 | 0.3 | >1000 |
| **STDEV** | 4.8 | 0.0 | 4.1 | 0.0 | 4.2 | 0.0 | 10.6 | 0.0 | 0.1 | 0.0 | 0.0 | 0.0 | 0.0 | 0.0 | 1.0 | 0.0 | 0.2 | 0.0 | 0.0 | 0.0 | 0.1 | 0.0 | 0.2 | 0.0 |
| **MAX** | 1.0 | >1000 | 4.0 | >1000 | 4.0 | >1000 | 4.0 | >1000 | 0.1 | >1000 | 0.2 | >1000 | 0.1 | >1000 | 0.1 | >1000 | 0.1 | >1000 | 0.1 | >1000 | 0.3 | >1000 | 0.1 | >1000 |
| **MIN** | 12.0 | >1000 | 12.0 | >1000 | 12.0 | >1000 | 25.0 | >1000 | 0.4 | >1000 | 0.3 | >1000 | 0.2 | >1000 | 2.0 | >1000 | 0.4 | >1000 | 0.1 | >1000 | 0.5 | >1000 | 0.5 | >1000 |
| **Gentamicin** |  |  |  |  |  |  |  |  |  |  |  |  |  |  |  |  |  |  |  |  |  |  |  |  |
| **MIC in mg/L** | 8.1 | >1000 | 14.4 | >1000 | 10.8 | >1000 | 22.0 | >1000 | 17.7 | >1000 | 14.3 | >1000 | 19.0 | >1000 | 8.0 | >1000 | 42.7 | >1000 | 10.7 | >1000 | 2.0 | >1000 | 2.0 | >1000 |
| **STDEV** | 5.0 | 0.0 | 7.4 | 0.0 | 2.5 | 0.0 | 5.2 | 0.0 | 6.7 | 0.0 | 9.7 | 0.0 | 5.2 | 0.0 | 0.0 | 0.0 | 23.9 | 0.0 | 4.6 | 0.0 | 0.0 | 0.0 | 0.0 | 0.0 |
| **MAX** | 2.0 | >1000 | 8.0 | >1000 | 8.0 | >1000 | 16.0 | >1000 | 12.0 | >1000 | 6.0 | >1000 | 16.0 | >1000 | 8.0 | >1000 | 16.0 | >1000 | 8.0 | >1000 | 2.0 | >1000 | 2.0 | >1000 |
| **MIN** | 12.5 | >1000 | 25.0 | >1000 | 12.5 | >1000 | 25.0 | >1000 | 25.0 | >1000 | 25.0 | >1000 | 25.0 | >1000 | 8.0 | >1000 | 62.0 | >1000 | 16.0 | >1000 | 2.0 | >1000 | 2.0 | >1000 |
| **Imipenem** |  |  |  |  |  |  |  |  |  |  |  |  |  |  |  |  |  |  |  |  |  |  |  |  |
| **MIC in mg/L** | 0.4 | >1000 | 0.4 | >1000 | 1.6 | >1000 | 0.4 | >1000 | 1.2 | >1000 | 1.7 | >1000 | 1.2 | >1000 | 0.9 | >1000 | 1.8 | >1000 | 2.0 | >1000 | 26.0 | >1000 | 26.0 | >1000 |
| **STDEV** | 0.4 | 0.0 | 0.4 | 0.0 | 1.4 | 0.0 | 0.5 | 0.0 | 0.3 | 0.0 | 1.2 | 0.0 | 0.3 | 0.0 | 0.6 | 0.0 | 1.4 | 0.0 | 0.0 | 0.0 | 8.7 | 0.0 | 8.7 | 0.0 |
| **MAX** | 0.1 | >1000 | 0.1 | >1000 | 0.3 | >1000 | 0.1 | >1000 | 1.0 | >1000 | 1.0 | >1000 | 1.0 | >1000 | 0.3 | >1000 | 0.3 | >1000 | 2.0 | >1000 | 16.0 | >1000 | 16.0 | >1000 |
| **MIN** | 0.8 | >1000 | 0.8 | >1000 | 3.0 | >1000 | 0.8 | >1000 | 1.5 | >1000 | 3.0 | >1000 | 1.5 | >1000 | 1.5 | >1000 | 3.0 | >1000 | 2.0 | >1000 | 31.0 | >1000 | 31.0 | >1000 |
|  |  |  |  |  |  |  |  |  |  |  |  |  |  |  |  |  |  |  |  |  |  |  |  |  |
|  |  |  |  |  |  |  |  |  |  |  |  |  |  |  |  |  |  |  |  |  |  |  |  |  |
|  | **LM EGDe** | | **LM MFPT3** | | **LM Scott A** | | **LM QC 1** | | **EC BW25113** | | **EC JW5503** | | **EC ATCC25922** | | **BC ATCC11778** | | **BC KSS** | | **PA ATCC** | | **PA K71** | | **PA K73** | |
| **Benzalkoniumchloride** | **culturable** | **VBNC** | **culturable** | **VBNC** | **culturable** | **VBNC** | **culturable** | **VBNC** | **culturable** | **VBNC** | **culturable** | **VBNC** | **culturable** | **VBNC** | **culturable** | **VBNC** | **culturable** | **VBNC** | **culturable** | **VBNC** | **culturable** | **VBNC** | **culturable** | **VBNC** |
| **MIC in mg/L** | 5.2 | 375.0 | 11.4 | 400.0 | 5.5 | 375.0 | 5.5 | 375.0 | 39.0 | 750.0 | 5.5 | 687.5 | 29.0 | 583.3 | 4.7 | 250.0 | 7.3 | 250.0 | 83.0 | 208.3 | 31.0 | 208.3 | 41.3 | 208.3 |
| **STDEV** | 1.8 | 144.3 | 1.9 | 136.9 | 1.0 | 144.3 | 1.0 | 144.3 | 12.9 | 288.7 | 1.0 | 375.0 | 14.6 | 381.9 | 2.3 | 0.0 | 4.2 | 0.0 | 36.4 | 72.2 | 0.0 | 72.2 | 17.9 | 72.2 |
| **MAX** | 2.0 | 250.0 | 8.0 | 250.0 | 4.0 | 250.0 | 4.0 | 250.0 | 25.0 | 500.0 | 4.0 | 250.0 | 16.0 | 250.0 | 2.0 | 250.0 | 4.0 | 250.0 | 62.0 | 125.0 | 31.0 | 125.0 | 31.0 | 125.0 |
| **MIN** | 6.0 | 500.0 | 12.5 | 500.0 | 6.0 | 500.0 | 6.0 | 500.0 | 50.0 | 1000.0 | 6.0 | 1000.0 | 50.0 | 1000.0 | 6.0 | 250.0 | 12.0 | 250.0 | 125.0 | 250.0 | 31.0 | 250.0 | 62.0 | 250.0 |
| **Bronopol** |  |  |  |  |  |  |  |  |  |  |  |  |  |  |  |  |  |  |  |  |  |  |  |  |
| **MIC in mg/L** | 51.7 | 500.0 | 72.7 | 1000.0 | 51.7 | 666.7 | 62.0 | 666.7 | 36.3 | 1000.0 | 51.7 | 1000.0 | 36.3 | 1000.0 | 8.7 | 500.0 | 6.7 | 666.7 | 2.0 | >1000 | 4.0 | 1000.0 | 4.0 | 1000.0 |
| **STDEV** | 17.9 | 0.0 | 47.9 | 0.0 | 17.9 | 288.7 | 0.0 | 288.7 | 23.5 | 0.0 | 17.9 | 0.0 | 23.5 | 0.0 | 7.0 | 0.0 | 2.3 | 288.7 | 0.0 | >1000 | 0.0 | 0.0 | 0.0 | 0.0 |
| **MAX** | 31.0 | 500.0 | 31.0 | 1000.0 | 31.0 | 500.0 | 62.0 | 500.0 | 16.0 | 1000.0 | 31.0 | 1000.0 | 16.0 | 1000.0 | 2.0 | 500.0 | 4.0 | 500.0 | 2.0 | 0.0 | 4.0 | 1000.0 | 4.0 | 1000.0 |
| **MIN** | 62.0 | 500.0 | 125.0 | 1000.0 | 62.0 | 1000.0 | 62.0 | 1000.0 | 62.0 | 1000.0 | 62.0 | 1000.0 | 62.0 | 1000.0 | 16.0 | 500.0 | 8.0 | 1000.0 | 2.0 | 0.0 | 4.0 | 1000.0 | 4.0 | 1000.0 |
| **Sodium azide** |  |  |  |  |  |  |  |  |  |  |  |  |  |  |  |  |  |  |  |  |  |  |  |  |
| **MIC in mg/L** | 145.7 | >1000 | 166.7 | >1000 | 125.0 | >1000 | 250.0 | >1000 | 833.3 | >1000 | 1000.0 | >1000 | 833.3 | >1000 | 62.0 | >1000 | 83.0 | >1000 | 166.7 | >1000 | 125.0 | >1000 | 41.3 | >1000 |
| **STDEV** | 95.7 | 0.0 | 72.2 | 0.0 | 0.0 | 0.0 | 0.0 | 0.0 | 288.7 | 0.0 | 0.0 | 0.0 | 288.7 | 0.0 | 0.0 | 0.0 | 36.4 | 0.0 | 72.2 | 0.0 | 0.0 | 0.0 | 17.9 | 0.0 |
| **MAX** | 62.0 | >1000 | 125.0 | >1000 | 125.0 | >1000 | 250.0 | >1000 | 500.0 | >1000 | 1000.0 | >1000 | 500.0 | >1000 | 62.0 | >1000 | 62.0 | >1000 | 125.0 | >1000 | 125.0 | >1000 | 31.0 | >1000 |
| **MIN** | 250.0 | >1000 | 250.0 | >1000 | 125.0 | >1000 | 250.0 | >1000 | 1000.0 | >1000 | 1000.0 | >1000 | 1000.0 | >1000 | 62.0 | >1000 | 125.0 | >1000 | 250.0 | >1000 | 125.0 | >1000 | 62.0 | >1000 |
| **TOMA chloride** |  |  |  |  |  |  |  |  |  |  |  |  |  |  |  |  |  |  |  |  |  |  |  |  |
| **MIC in mg/L** | 2.3 | 375.0 | 1.3 | 375.0 | 1.2 | 333.3 | 1.7 | 333.3 | 6.7 | 625.0 | 4.0 | 625.0 | 4.0 | 333.3 | 0.3 | 416.7 | 0.4 | 666.7 | 125.0 | 1000.0 | 16.0 | 416.7 | 8.0 | 416.7 |
| **STDEV** | 1.5 | 144.3 | 0.6 | 144.3 | 0.8 | 144.3 | 0.6 | 144.3 | 2.3 | 433.0 | 0.0 | 433.0 | 0.0 | 144.3 | 0.2 | 144.3 | 0.2 | 288.7 | 0.0 | 0.0 | 0.0 | 144.3 | 0.0 | 144.3 |
| **MAX** | 1.0 | 250.0 | 1.0 | 250.0 | 0.5 | 250.0 | 1.0 | 250.0 | 4.0 | 250.0 | 4.0 | 250.0 | 4.0 | 250.0 | 0.1 | 250.0 | 0.1 | 500.0 | 125.0 | 1000.0 | 16.0 | 250.0 | 8.0 | 250.0 |
| **MIN** | 4.0 | 500.0 | 2.0 | 500.0 | 2.0 | 500.0 | 2.0 | 500.0 | 8.0 | 1000.0 | 4.0 | 1000.0 | 4.0 | 500.0 | 0.5 | 500.0 | 0.5 | 1000.0 | 125.0 | 1000.0 | 16.0 | 500.0 | 8.0 | 500.0 |
